# Radical total pelvic exenteration with concomitant right nephrectomy in the management of recurrent endometrioid ovarian adenocarcinoma: A case report and literature review

**DOI:** 10.1002/ccr3.9148

**Published:** 2024-07-03

**Authors:** Raghavskandhan Ramachandran, Sabina Nistor, William Gietzmann, Nicholas Symons, Hooman Soleymani majd

**Affiliations:** ^1^ Medical Sciences Division University of Oxford Oxford UK; ^2^ Department of Gynaecology Oncology Churchill Hospital, Oxford University Hospitals NHS Foundation Trust Oxford UK; ^3^ Department of Urology Oxford University Hospitals NHS Foundation Trust Oxford UK; ^4^ Department of Colorectal Surgery Oxford University Hospitals NHS Foundation Trust Oxford UK; ^5^ Nuffield Department of Women's and Reproductive Health University of Oxford Oxford UK

**Keywords:** bowel diversion, exenteration, neoplasm recurrence, ovarian cancer, pelvic surgery, urinary diversion

## Abstract

Endometrioid ovarian adenocarcinoma is a common subtype of epithelial ovarian cancer that can arise on a background of endometriosis. Maximal cytoreductive effort with an aim to remove all macroscopic disease (achieve R0) is the single independent prognostic factor for survival. Complex multidisciplinary surgeries may be required in order to achieve this.

## 1 BACKGROUND

Ovarian cancer is a heterogenous malignancy with multiple histological subtypes. The most common form of ovarian cancer is epithelial ovarian cancer, of which the serous subtype is most common. The second most common, accounting for approximately 10% of epithelial ovarian cancers, is endometrioid carcinoma.^1^ As a result of the rarer nature of ovarian endometrioid carcinoma, research and understanding of this subtype, its presentation, management, and prognosis, are limited. Cytoreductive surgery remains the mainstay treatment for this subtype of ovarian cancer, and the extent of tumor resection has been suggested to be a major prognostic factor.^2^, ^3^, ^4^

Here we present the case of a woman in her late 40's with pelvic recurrence of endometrioid ovarian cancer. The patient has consented for this case report to be written and published. The initial histopathology report graded the tumor as grade 1, without any evidence of clear cells, an indicator of aggressiveness. The cancer was International Federation of Gynecology and Obstetrics (FIGO) stage IC2, restricted to the ovaries and on a background of atypical endometriosis. This case highlights the surgical complexity of optimal cytoreductive surgery in recurrent endometrioid ovarian cancer, best achieved by a multidisciplinary approach.

## 2 CASE PRESENTATION

This woman in her late 40's, with a parity of 1, had a medical history of asthma and a BMI of 37. She was referred from the cancer unit to our tertiary cancer centre with a suspected recurrence of ovarian cancer. The patient had a history of grade 1 endometrioid ovarian cancer, FIGO Stage 1C2, treated 4 years prior to the referral in a cancer unit with surgery (total abdominal hysterectomy, bilateral salpingo‐oopherectomy, omentectomy, and peritoneal washings) followed by 5 cycles of chemotherapy (Carboplatin/Paclitaxel). A sixth cycle of chemotherapy was not administered due to toxicity. The patient was followed up with four monthly appointments in the first 2 years post surgery, then six monthly appointments. At a follow‐up appointment in the third year post surgery, the patient reported mild abdominal discomfort, intermittent lower back pain and had a CA 125 of 42 ku/L. A CT AP was requested and this identified a complex pelvic mass, of approximately seven centimeter in maximum diameter. The imaging was discussed at the Oxford multidisciplinary meeting (MDT) and a decision was made for a diagnostic laparoscopy. A tissue biopsy was obtained and was in keeping with an atypical endometrioma. The patient had GnRH analogues for 6 months followed by serial imaging.

## 3 INVESTIGATIONS

A CT abdomen and pelvis identified a 12 cm pelvic mass with thickening of the wall in keeping with malignant change. The suspicion of malignancy was confirmed by magnetic resonance imaging (MRI) and positron emission tomography (PET CT) scans which showed a part‐cystic, part‐solid mass with restricted diffusion and high FDG avidity, respectively. The preoperative MRI pelvis also described adhesions between the pelvic mass and the bowel. Imaging did not suggest any lymph node involvement or distant metastatic disease.

The right ureter was noted to be obstructed by the mass, leading to hydronephrosis and marked right kidney atrophy. A dimercaptosuccinic acid (DMSA) scan revealed minimal right kidney function.

The Oxford network MDT supported a two‐stage surgical procedure; firstly an exploratory laparoscopy to assess resectability of disease with intention to achieve R0, followed by radical exenterative surgery if suitable.

## 4 DIFFERENTIAL DIAGNOSIS

Endometrioid ovarian cancer may arise from an atypical endometrioma. Distinguishing these two pathologies on imaging may sometimes be challenging due to a number of factors: the ovarian parenchyma adjacent to an endometriotic cyst can be mistaken for an enhancing solid malignant nodule and deep invasive endometriosis of the recto‐sigmoid colon can mimic the “mushroom cap” sign associated with invasive malignancy (30).

## 5 TREATMENT

The patient was extensively counseled preoperatively, as per the Oxford pelvic exenterative team protocol. Ahe had multiple consultations with the gynecology oncology, urology, and colorectal teams. The cancer nurse specialist (CNS) played a central role in supporting her throughout this process. The stoma nurses were also involved as part of stoma planning. The risks and benefits of this surgery, the extent of the surgery, and also the option of doing nothing were explored. Early and late surgical complications were discussed and realistic expectations were set regarding the recovery period, including the need for permanent stomas. The patient was motivated to pursue surgical management. She had a full anaesthetic work‐up prior to surgery. Her diet, level of activity and psychological status were assessed as part of the prehabilitation programme. She was encouraged to maintain a healthy and active lifestyle before the operation.

The final surgical plan was agreed at a surgical MDT with the gynecology oncology, urology, and colorectal teams.

### 5.1 Surgical procedure

#### 5.1.1 Preparation and initial steps

**FIGURE 1:**
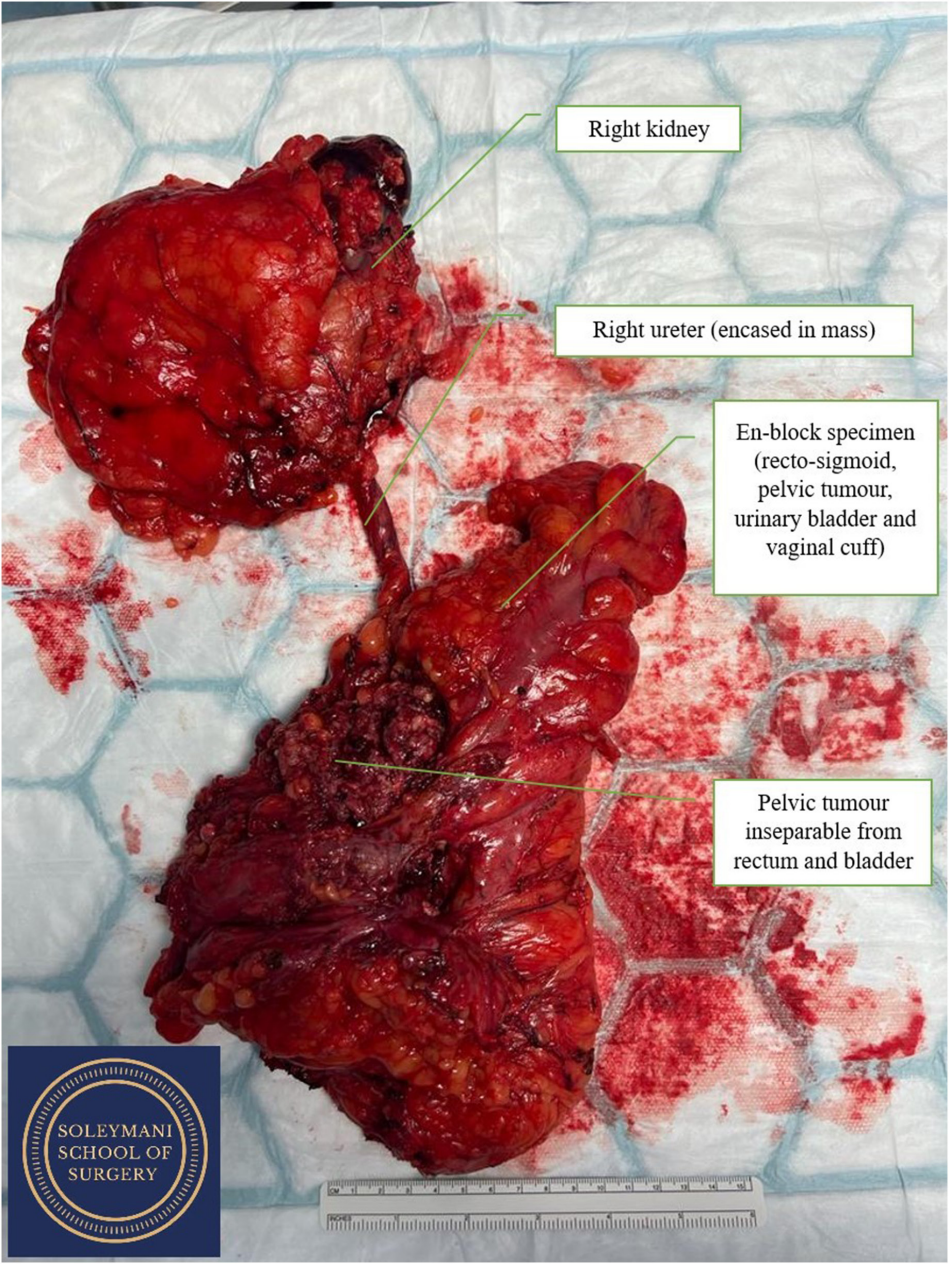
En bloc specimen containing right kidney, right ureter, recto‐sigmoid colon, pelvic tumor, urinary bladder, and vaginal cuff.

**FIGURE 2:**
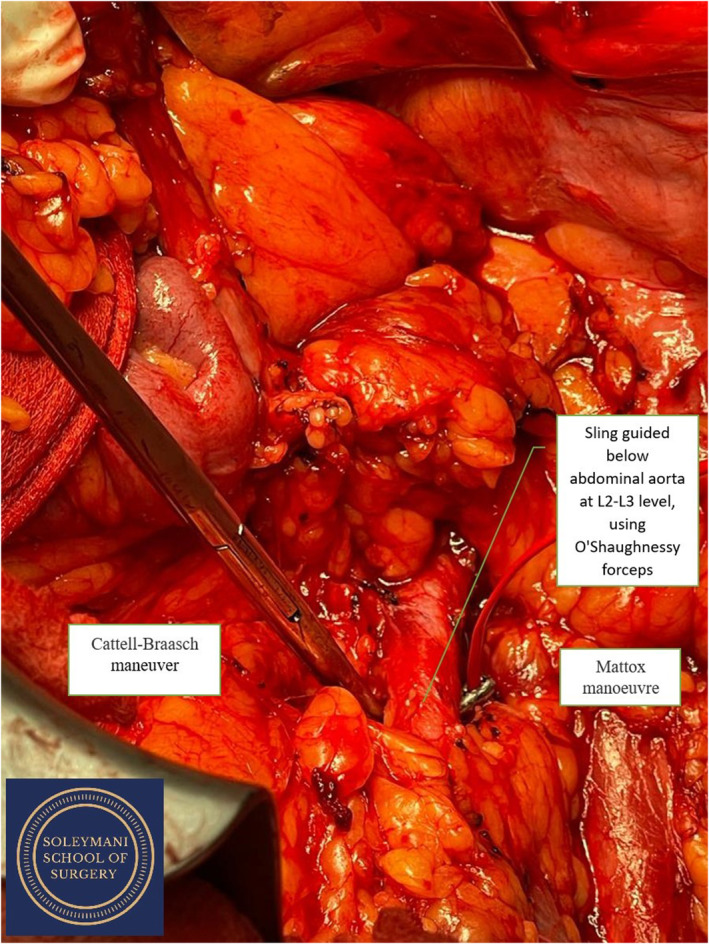
Sling guided below abdominal aorta after bowel mobilization using Cattell‐Braasch and Mattox maneuvers.

**FIGURE 3:**
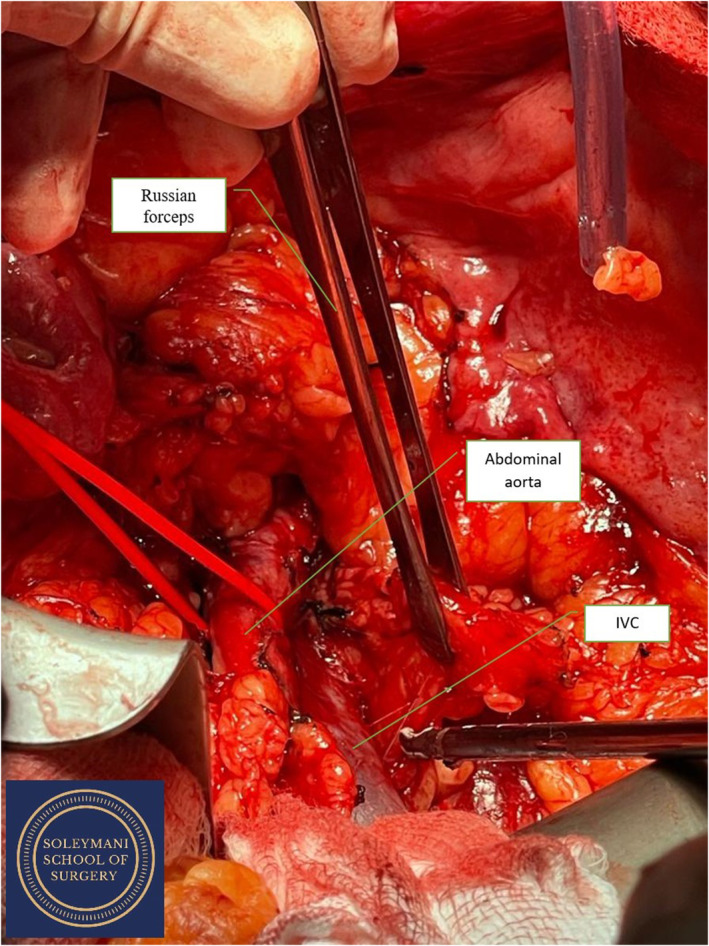
Abdominal aorta (red sling) and IVC.

The surgery was conducted as a joint procedure between the gynecology oncology, urology and colorectal teams. The patient was first cleaned, draped, catheterised, and positioned with her legs in a modified Llyod‐Davies position with flow‐trons on. A midline xiphi‐pubic laparotomy was performed and the abdomen was opened in layers with a handheld diathermy and Lahey dissecting forceps. The ascending colon and the liver were then mobilized to gain access to the right kidney for the nephrectomy. (Figures 1, 2, 3).

#### 5.1.2 Right kidney mobilization

The right nephrectomy was conducted first as the right kidney had been chronically obstructed by the tumor and appeared atrophic. The ureter was slung, and lower pole, upper pole and lateral attachments were dissected. The renal artery, vein, and gonadal vein were then all identified and transected, enabling mobilization of the right kidney. The ureter was followed to the level of the mass.

#### 5.1.3 Assessment of mass

A 15 × 15cm pelvic mass was identified in the Pouch of Douglas. The mass was seen to be inseparable from the urinary bladder and recto‐sigmoid colon and was in close proximity to the left vesico‐ureteric junction. The right ureter was encased in the mass. As the tumor was friable, a fragment detached when mobilization was attempted. This tissue was sent for frozen section. The frozen section report suggested the tumor was a grade 1 adenocarcinoma, either endometrioid, or endometrioid with mucinous component. Pelvic side walls were opened bilaterally, and para‐rectal, para‐vesical, cave of Retzius and Latzko spaces developed. Subsequent discussion among the four consultant surgeons present finally led to the conclusion that a total exenteration was required to achieve complete tumor clearance.

#### 5.1.4 Pelvic exenteration

**FIGURE 4:**
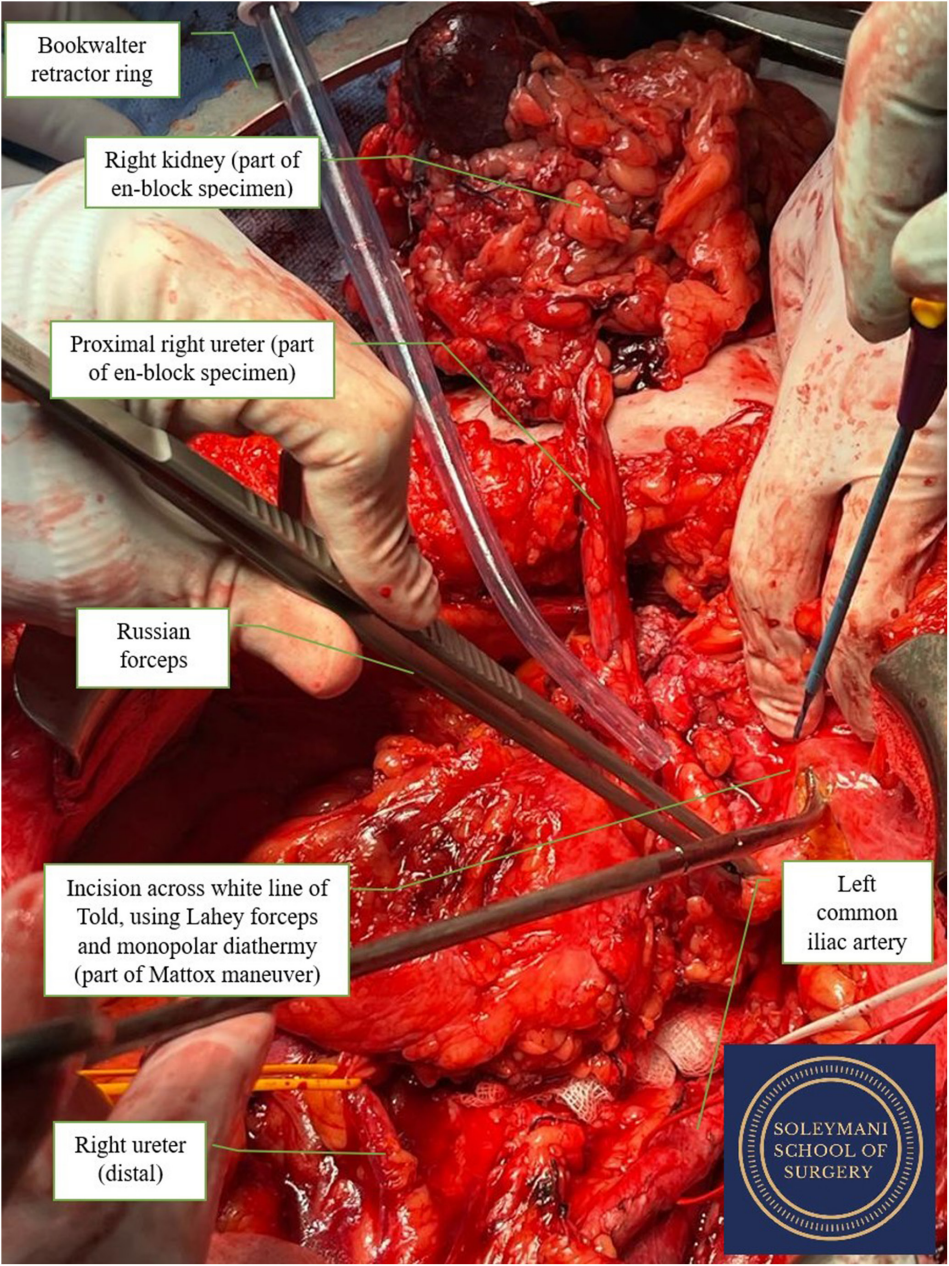
En bloc specimen after right nephrectomy and mobilization of right ureter; Mattox maneuver.

The pelvic exenteration began with the division of the sigmoid colon above the tumor and dissection of the total mesorectal excision (TME) plane to the pelvic floor. A radical cystectomy was performed, and the bladder was mobilized. Subsequent vaginal division and low rectal division then allowed for en‐bloc removal of the entire specimen consisting of right kidney, right ureter, urinary bladder, pelvic mass, and recto‐sigmoid colon and vaginal cuff. (Figure 4).

#### 5.1.5 Colonic conduit and end colostomy

Following the pelvic exenteration, a colonic conduit and end colostomy were required. First, the splenic flexure was mobilized. The proximal descending colon was then divided, and the distal section used to create the colonic conduit with the left ureter (Bricker procedure). The colonic conduit and proximal segment of the descending colon were matured through the left abdomen and two stomas produced in a vertical orientation.

#### 5.1.6 Closure

Before closure, bulky aorto‐caval lymph nodes that had been noticed earlier were removed. The pelvis was washed out and a Jackson‐Pratt drain inserted. The abdomen was then closed in layers. The total blood loss was 1500mLs.

## 6 OUTCOME AND FOLLOW‐UP

Due to the complex and extensive nature of the surgery, the patient was admitted to the Intensive Care Unit post‐operatively and stayed as an inpatient for the next 11 days before discharge. There were no significant complications in this period. The patient was readmitted 4 weeks post surgery with superficial wound dehiscence and urosepsis, which were managed with intravenous antibiotics (grade 1 Clavier Dindo complications). She recovered well following this admission. The final histopathology report of the removed section revealed the tumor was matted to the bladder and the rectum, but there was no evidence of invasion of the wall of these structures. The en‐bloc specimen had clear margins. The aorto‐caval lymph nodes removed were normal with no evidence of metastatic carcinoma. These findings were discussed in MDT, along with immunohistochemistry results, and the ultimate decision was that adjuvant chemotherapy or radiotherapy was not indicated. This decision was subsequently conveyed to the patient who had made a full recovery post surgery. Benchmark imaging (CT chest, abdomen, pelvis and MRI pelvis) performed 4 months following the total exenteration were unremarkable.

## 7 DISCUSSION

Endometrioid carcinomas are the second most common epithelial ovarian carcinomas. In contrast to the more common serous carcinomas, endometrioid carcinomas present earlier, at a younger age and have better long‐term outcomes.^5^, ^6^, ^7^ Several studies have suggested that endometrioid carcinomas have higher rates of both 5‐year overall survival (80.6%) and progression‐free survival (68%) compared to other ovarian cancer subtypes. Additionally, they have a lower recurrence rate, especially at lower grades.^1^, ^6^ Relapse patterns appear to further differentiate endometrioid carcinomas from serous carcinomas, with endometrioid carcinomas having a much higher proportion, at nearly 50%, recurring solely in the pelvis, while serous carcinoma relapse tends to be much more diffuse.^6^ Here we discuss an interesting, rare presentation of a pelvic recurrence of low grade endometrioid carcinoma.

Endometriosis is a risk factor for the development of ovarian cancer, mostly specific subtypes including endometrioid and clear cell carcinomas. Patients with endometrioid ovarian adenocarcinoma arising from endometriosis tend to present at a younger age, lower stage, and lower grade than those without associated endometriosis.^8^ The transformation from benign to atypical endometriosis to endometriosis‐associated ovarian cancer involves a combination of: oxidative stress, inflammation, molecular genomic alterations and hyperoestrogenism. Some of the molecular abnormalities encountered in endometriosis‐associated ovarian cancer include: the activation of oncogenic KRAS and PI3 K pathways and the inactivation of tumor suppressor genes PTEN and AT‐Rich Interaction Domain 1A (ARID1A). Of the key mutations involved in the malignant transformation and progression, including ARID1A mutations, some have the potential to be effective chemotherapy targets.^9^, ^10^

In a patient with endogenic hyperoestrogenism related to obesity, a new pelvic mass diagnosed 3 years from the initial surgery may represent, rather than a recurrence, a de novo lesion progressing from benign endometriosis to atypical endometriosis and then endometrioid ovarian adenocarcinoma.

Other important pathologies have also been noted to be associated with ovarian endometrioid carcinoma that should be understood and considered, including endometrial cancer. The rare presentation of primary tumors in both the endometrium and ovary in synchronous endometrial and ovarian carcinoma, is recognized to be a separate entity to either pathology with different prognoses and treatment implications.^11^, ^12^, ^13^ These tumors were previously thought to be synchronous independent tumors, however molecular analysis has established that they have a common clonal origin.^14^ The 2023 FIGO staging of endometrial cancer, which incorporates molecular findings, classifies these tumors as stage IA3 when certain criteria are met: unilateral disease, no capsular spread, less than 50% myometrial invasion, absence of substantial/extensive lympho‐vascular space invasion (LVSI). These tumors have a better prognosis and do not require adjuvant chemotherapy.^15^

The Carboplatin/Paclitaxel regimen as adjuvant treatment has not been proven to result in survival benefit for low‐grade endometrioid ovarian cancer.^16^ Current National Comprehensive Cancer Network (NCCN) guidelines^17^ for grade 1 endometroid ovarian carcinoma are Carboplatin/Paclitaxel or hormonal treatment, such as aromatase inhibitors, leuprolide acetate, tamoxifen. Novel, biomarker‐driven therapies, are currently being investigated for this histological subtype: Bouquet (NCT04931342 GOG‐3051) is a multicentre clinical trial which is currently recruiting patients with persistent or recurrent low‐grade endometrioid ovarian cancer and other rare ovarian tumors that are not amenable to curative surgery.

Until new treatments options are identified, surgery with maximal cytoreductive effort remains the mainstay treatment for this histological subtype of ovarian cancer.^18^, ^19^, ^20^, ^21^, ^22^, ^23^, ^24^, ^25^, ^26^ Achieving resection of all macroscopic disease (R0) is the single independent factor for survival. In their meta‐analysis including 6885 patients with advanced ovarian cancer, Bristow et al (2023) have shown there is a 5.5% increase in median survival time with each 10% increase in maximal cytoreduction.^2^

In the case we presented, a radical total exenteration was required to achieve R0. A right nephrectomy was performed to remove the right kidney, which was nonfunctioning. The Bricker technique for urinary diversion involved spatulation of the remaining ureter and anastomosis to a segment of descending colon used as a conduit. Total cystectomy with urinary diversion is a standard procedure in the context of anterior or total exenterations.^27^, ^28^ The use of a segment of descending colon should be considered in patients undergoing end colostomy, to avoid the need for primary small bowel anastomosis.

In conclusion, ovarian endometrioid carcinoma of the ovary is an uncommon histological subtype of ovarian carcinoma for which the mainstay of treatment is surgery. Cytoreductive effort should be maximized to achieve R0, especially in young patients. The complexity of the operation should not be a deterrent factor if surgery is carried out in a multidisciplinary environment with robust clinical governance.

## AUTHOR CONTRIBUTIONS

**Raghavskandhan Ramachandran:** Conceptualization; writing – original draft; writing – review and editing. **Sabina Nistor:** Writing – original draft; writing – review and editing. **William Gietzmann:** Writing – original draft; writing – review and editing. **Nicholas Symons:** Writing – original draft; writing – review and editing. **Hooman Soleymani majd:** Conceptualization; project administration; writing – original draft; writing – review and editing.

## FUNDING INFORMATION

None.

## CONFLICT OF INTEREST STATEMENT

The authors have no conflicts of interest.

## CONSENT

Written informed consent was obtained from the patient to publish this report in accordance with the journal's patient consent policy.

## Data Availability

Data sharing not applicable to this article as no datasets were generated or analysed during the current study.

## References

[1] Pecorino B , Lagana AS , Chiantera V , et al. Progression free survival, overall survival, and relapse rate in Endometrioid ovarian cancer and synchronous endometrial‐ovarian Endometrioid cancer (SEO‐EC): results from a large retrospective analysis. Medicina. 2022;58(12):1706. doi:10.3390/medicina58121706 36556908 PMC9784653

[2] Bristow RE , Tomacruz RS , Armstrong DK , Trimble EL , Montz FJ . Survival effect of maximal cytoreductive surgery for advanced ovarian carcinoma during the platinum era: a meta‐analysis. J Clin Oncol. 2023;41(25):4065‐4076. doi:10.1200/JCO.22.02765 37643543

[3] Ledermann JA , Raja FA , Fotopoulou C , et al. Newly diagnosed and relapsed epithelial ovarian carcinoma: ESMO clinical practice guidelines for diagnosis, treatment and follow‐up. Ann Oncol. 2018;29(4):259. doi:10.1093/annonc/mdy157 30285216

[4] Leitao MM Jr , Chi DS . Surgical management of recurrent ovarian cancer. Semin Oncol. 2009;36(2):106‐111. doi:10.1053/j.seminoncol.2008.12.002 19332245

[5] Fortner RT , Trewin‐Nybraten CB , Paulsen T , Langseth H . Characterization of ovarian cancer survival by histotype and stage: a nationwide study in Norway. Int J Cancer. 2023;153(5):969‐978. doi:10.1002/ijc.34576 37226635

[6] Bouchard‐Fortier G , Panzarella T , Rosen B , Chapman W , Gien LT . Endometrioid carcinoma of the ovary: outcomes compared to serous carcinoma after 10 years of follow‐up. J Obstet Gynaecol Can. 2017;39(1):34‐41. doi:10.1016/j.jogc.2016.10.006 28062021

[7] Li S , Zhu Z . Chemotherapy is not necessary for early‐stage serous and endometrioid ovarian cancer after undergoing comprehensive staging surgery. J Ovarian Res. 2020;13(1):91. doi:10.1186/s13048-020-00694-9 32772926 PMC7416408

[8] Paik ES , Kim TJ , Choi CH , Kim BG , Bae DS , Lee JW . Clinical outcomes of patients with clear cell and endometrioid ovarian cancer arising from endometriosis. J Gynecol Oncol. 2018;29(2):e18. doi:10.3802/jgo.2018.29.e18 29400011 PMC5823979

[9] Maeda D , Shih IM . Pathogenesis and the role of ARID1A mutation in endometriosis‐related ovarian neoplasms. Adv Anat Pathol. 2013;20(1):45‐52. doi:10.1097/PAP.0b013e31827bc24d 23232571 PMC3523307

[10] Ayhan A , Mao TL , Seckin T , et al. Loss of ARID1A expression is an early molecular event in tumor progression from ovarian endometriotic cyst to clear cell and endometrioid carcinoma. Int J Gynecol Cancer. 2012;22(8):1310‐1315. doi:10.1097/IGC.0b013e31826b5dcc 22976498 PMC3460070

[11] Makris GM , Manousopoulou G , Battista MJ , Salloum I , Chrelias G , Chrelias C . Synchronous endometrial and ovarian carcinoma: a case series. Case Rep Oncol. 2017;10(2):732‐736. doi:10.1159/000479501 28878658 PMC5582525

[12] Singh N . Synchronous tumours of the female genital tract. Histopathology. 2010;56(3):277‐285. doi:10.1111/j.1365-2559.2009.03367.x 20459528

[13] Le Thanh V , Bell R , Symons N , Soleymani MH . The role of multidisciplinary team and stepwise pelvic devascularization to minimize blood loss during total pelvic exenteration for patients refusing blood transfusion. Clin Case Reports. 2023;11(9):e7689. doi:10.1002/ccr3.7689 PMC10500049 37720708

[14] Anglesio MS , Wang YK , Maassen M , et al. Synchronous endometrial and ovarian carcinomas: evidence of Clonality. J Natl Cancer Inst. 2016;108(6):djv428. doi:10.1093/jnci/djv428 26832771

[15] Berek JS , Matias‐Guiu X , Creutzberg C , et al. Endometrial cancer staging subcommittee, FIGO Women's cancer committee FIGO staging of endometrial cancer: 2023. Int J Gynaecol Obstet. 2023;162(2):383‐394. doi:10.1002/ijgo.14923 37337978

[16] Swift BE , Covens A , Mintsopoulos V , et al. The effect of complete surgical staging and adjuvant chemotherapy on survival in stage I, grade 1 and 2 endometrioid ovarian carcinoma. Int J Gynecol Cancer. 2022;32(4):525‐531. doi:10.1136/ijgc-2021-003112 34969829

[17] Referenced with permission from the NCCN Clinical Practice Guidelines in Oncology (NCCN Guidelines®) for Ovarian Cancer V.2.2023. © National Comprehensive Cancer Network, Inc. 2023 All rights reserved. Accessed September 18, 2023. To view the most recent and complete version of the guideline, go online to NCCN.org

[18] Addley S , Alazzam M , Johnson C , Soleymani MH . Rectovaginal extragastrointestinal stromal tumour (EGIST): an additional entity to be considered in the differential diagnosis of tumours of the rectovaginal septum. BMJ Case Rep. 2021;14(3):e237669. doi:10.1136/bcr-2020-237669 PMC7942270 33685909

[19] Soleymani Majd H , Collins SL , Addley S , et al. The modified radical peripartum cesarean hysterectomy (Soleymani‐Alazzam‐Collins technique): a systematic, safe procedure for the management of severe placenta accreta spectrum. Am J Obstet Gynecol. 2021;225(2):175 e1‐175 e10. doi:10.1016/j.ajog.2021.03.014 33716074

[20] Cowdell I , Smyth SL , Eltawab S , Soleymani MH . Radical abdomino‐pelvic surgery in the management of uterine carcinosarcoma with concomitant para‐aortic lymphadenopathy metastasising from anal carcinoma. BMJ Case Rep. 2022;15(11):e252233. doi:10.1136/bcr-2022-252233 PMC9716812 36450419

[21] Khan M , Eltawab S , Gietzmann W , Soleymani MH . Laterally extended endopelvic resection as part of the surgical management of disseminated retroperitoneal leiomyomatosis mimicking low‐grade sarcoma in a patient with a solitary kidney. BMJ Case Rep. 2023;16(6):e254660. doi:10.1136/bcr-2023-254660 PMC10254902 37263674

[22] Cowie P , Eastwood B , Smyth S , Soleymani MH . Atypical presentation of intravascular leiomyomatosis mimicking advanced uterine sarcoma: modified laterally extended endopelvic resection with preservation of pelvic neural structures. BMJ Case Rep. 2021;14(9):e244774. doi:10.1136/bcr-2021-244774 PMC8449947 34531237

[23] Guerrisi R , Smyth SL , Ismail L , Horne A , Ferrari F , Soleymani MH . Approach to radical hysterectomy for cervical cancer in pregnancy: surgical pathway and ethical considerations. J Clin Med. 2022;11(24):7352. doi:10.3390/jcm11247352 36555968 PMC9781163

[24] Smyth SL , Siddiqi A , Athanasou N , Whitwell D , Soleymani MH . Adamantinoma: a review of the current literature. J Bone Oncol. 2023;41:100489. doi:10.1016/j.jbo.2023.100489 37408735 PMC10318513

[25] Smyth S , Addley S , Alazzam M , Soleymani MH . Adamantinoma: metastatic disease masquerading as a gynaecological malignancy. BMJ Case Rep. 2021;14(6):e241615. doi:10.1136/bcr-2021-241615 PMC8183221 34083182

[26] Tozzi R , Hardern K , Gubbala K , Garruto Campanile R , Soleymani MH . En‐bloc resection of the pelvis (EnBRP) in patients with stage IIIC‐IV ovarian cancer: a 10 steps standardised technique. Surgical and survival outcomes of primary vs. interval surgery. Gynecol Oncol. 2017;144(3):564‐570. doi:10.1016/j.ygyno.2016.12.019 28073597

[27] Addley S , Soleymani MH . Laparoscopic resection of single site pelvic side wall recurrence 6 years after stage IIIc high grade serous primary peritoneal cancer. Gynecol Oncol Rep. 2021;36:100709. doi:10.1016/j.gore.2021.100709 33718559 PMC7909385

[28] Cerdeira AS , Ismail L , Moore N , George B , Majd HS . Retroperitoneal leiomyomatosis: a benign outcome of power morcellation with potentially serious consequences. Lancet. 2022;399(10324):554. doi:10.1016/S0140-6736(22)00005-8 35123695

